# “Missing mutations” in MPS I: Identification of two novel copy number variations by an *IDUA*‐specific *in house* MLPA assay

**DOI:** 10.1002/mgg3.615

**Published:** 2019-07-18

**Authors:** Amir Jahic, Sven Günther, Nicole Muschol, Barbro Fossøy Stadheim, Øivind Braaten, Hanne Kjensli Hyldebrandt, Gé‐Ann Kuiper, Karen Tylee, Frits A. Wijburg, Christian Beetz

**Affiliations:** ^1^ Institute of Clinical Chemistry and Laboratory Diagnostics Jena University Hospital Jena Germany; ^2^ Institute of Laboratory Medicine Clinical Chemistry and Pathobiochemistry Charité – Universitätsmedizin Berlin Berlin Germany; ^3^ Department of Pediatrics University Medical Center Hamburg‐Eppendorf Hamburg Germany; ^4^ Department of Clinical Genetics Oslo University Hospital Oslo Norway; ^5^ Pediatric Metabolic Diseases Amsterdam UMC University of Amsterdam Academic Medical Center (AMC) Amsterdam Netherlands; ^6^ Manchester Center for Genomic Medicine St Mary's Hospital Manchester UK; ^7^ Centogene AG Rostock Germany

**Keywords:** copy number variations, deletion, duplication, *IDUA*, MLPA

## Abstract

**Background:**

Mucopolysaccharidosis type I (MPS I) is a rare, recessively inherited lysosomal storage disorder, characterized by progressive multi‐systemic disease. It is caused by a reduced or absent alpha‐l iduronidase (IDUA) enzyme activity secondary to biallelic loss‐of‐function variants in the *IDUA*. Over 200 causative variants in *IDUA* have been identified. Nevertheless, there is a fraction of MPS I patients with only a single mutated *IDUA* allele detectable.

**Methods:**

As genetic testing of MPS I is usually based on sequencing methods, copy number variations (CNVs) in *IDUA* can be missed and therefore presumably remain underdiagnosed. The aim of this study was the detection of CNVs using an *IDUA*‐specific *in house* multiplex ligation‐dependent probe amplification (MLPA) assay.

**Results:**

A total of five unrelated MPS I patient samples were re‐analyzed after only a single heterozygous *IDUA* mutation c.979G>C (p.A327P), c.1469T>C (p.L490P), c.1598C>G (p.P533R), c.1205G>A (p.W402X), c.973‐7C>G (p.?) could be identified. We detected a novel splice site variant c.973‐7C>G (p.?), as well as two novel CNVs, a large deletion of *IDUA* exon 14 and 3’UTR c.(1828 + 1_1829‐1)_(*1963_?)del, and a large duplication extending from *IDUA* exon 2 to intron 12 c.(157 + 1_158‐1)_(1727 + 1_1728‐1)dup.

**Conclusion:**

Together with the CNVs we previously identified, a total of four pathogenic *IDUA* CNVs have now been reported.

## 1 INTRODUCTION

Mucopolysaccharidosis type I (MPS I) is a lysosomal storage disorder caused by biallelic loss‐of‐function variants in the *IDUA* (MIM #252800). Pathogenic *IDUA* variants lead to the deficiency of lysosomal alpha‐l iduronidase (IDUA; EC 3.2.1.76), a hydrolase involved in the catabolism of glycosaminoglycans (GAGs) dermatan and heparan sulfate. Reduced or absent IDUA enzyme activity results in the lysosomal accumulation of GAGs and the onset of pathology in specific cells, tissues, and organs (Poletto, Pasqualim, Giugliani, Matte, & Baldo, 12; Scott et al., 14). There are three clinical subtypes of MPS I: Hurler syndrome (MPS I H, MIM #607014; severe), Hurler‐Scheie syndrome (MPS I H/S, MIM #607015; intermediate), and Scheie syndrome (MPS I S, MIM #607016; attenuated). According to the severity of the disease, clinical signs and symptoms of MPS I are present in the first decade of life, including organomegaly, obstructive airway disease, heart disease, skeletal deformities, growth retardation, neurological complications, and severe mental retardation (Beck et al., 1; Cleary & Wraith, 7). However, the phenotypical spectrum is continuous and the classification is frequently complex (Poletto et al., 12). The clinical diagnosis of MPS I is confirmed biochemically based on elevated GAGs in urine, and reduced or absent IDUA activity in leucocytes or skin fibroblasts (Beesley et al., 2; Scott et al., 15). While clinical and biochemical abnormalities are present, genetic background still remains unresolved in a fraction of MPS I patients (Bunge et al., 5; Ghosh et al., 8; Scott et al., 15).

The spectrum of causative genetic variants in *IDUA* is highly heterogeneous as more than 200 single‐gene defects have been reported, including missense, nonsense, splice site, insertions, as well as small deletions and duplications (Bertola et al., 3). Recently we described a novel mutational mechanism for MPS I: two distinct large *IDUA*‐deleterious copy number variations (CNVs) detected by an in house *multiplex ligation‐dependent probe amplification* (MLPA) approach (Breen et al., 4).

In this study we used the same copy number screening tool as well as the Sanger‐based sequencing to re‐analyze five unrelated MPS I DNA samples, which were initially tested positive for a pathogenic variant on a single *IDUA* allele.

## 2 MATERIALS AND METHODS

### 2.1 Ethical compliance

The study was approved by the Ethics Committee of the Faculty of Medicine of Friedrich‐Schiller‐University Jena (reference number: 2018‐1107).

### 2.2 Patients

Following publication of the first two *IDUA* deletions and introduction of an *IDUA* MLPA assay (Breen et al., 4), the corresponding author was contacted by several clinicians who had MPS I patients in whom only a single mutated *IDUA* allele could be identified. DNA samples with consent for extended genetic workup were eventually enrolled. Table 1 summarizes the geographic, clinical, biochemical, and genetic backgrounds of the patients investigated.

**Table 1:** *IDUA* variants identified in this study. Nomenclature for cDNA and protein is based on reference sequences NM_000203.4 and NP_000194.2, respectively

Patient	1st allele variant		2nd allele variant		IDUA enzyme		Clinical subtype	Origin
	DNA	Protein	DNA	Protein	Activity	Ref. range		
**I**	**c.973‐7C>G**	n/a	n.i.	n.i.	0.00	0.27 – 9.00	H	Germany/Italy
**II**	c.979G>C	p.A327P	n.i.	n.i.	0.01	ctrl sample	H	Germany
**III**	c.1205G>A	p.W402X	n.i.	n.i.	0.60	14.0 – 40.0	S	Holland
**IV**	c.1469T>C	p.L490P	c.(1828 + 1_1829‐1)_(*1963_?)del	n/a	0.00	0.14 – 0.35	H	Pakistani/Norway
**V**	c.1598C>G	p.P533R	**c.(157 + 1_158‐1)_(1727 + 1_1728‐1)dup**	n/a	n.a.	n.a.	H	United Kingdom

n/a, not applicable; n.a., not available; n.i., not identified; variants highlighted in bold: novel variants; leucocyte IDUA enzyme activity has been measured in: nmol/mg*hour (patient I and III), mU (patient II), μmol/mg*hour (patient IV); ctrl sample, control blood sample; ref. range, reference range, H, Hurler, S, Scheie.

### 2.3 Genetic analyses

The *IDUA* coding sequence plus >50 nucleotides of neighboring UTR or intronic sequence was amplified from genomic DNA (primers available upon request). PCR products were gel‐purified and Sanger‐sequenced from both directions using a commercial service (Macrogen Europe, Amsterdam, The Netherlands).

*IDUA*‐specific MLPA was based on the synthetic kit presented by us previously (Breen et al., 4). In the frame of the study, this assay was extended with additional MLPA probes; corresponding oligonucleotides were purchased from Biolegio (Nijmegen, The Netherlands). Supporting Information Table S1AB provides all target sequences and product sizes. Variants were described at cDNA and protein level using reference sequences NM_000203.4 and NP_000194.2, respectively.

## 3 RESULTS

### 3.1 Sanger‐sequencing detects a single heterozygous IDUA variant in five unrelated patients with a clinical diagnosis of MPS I

**Figure 1:**
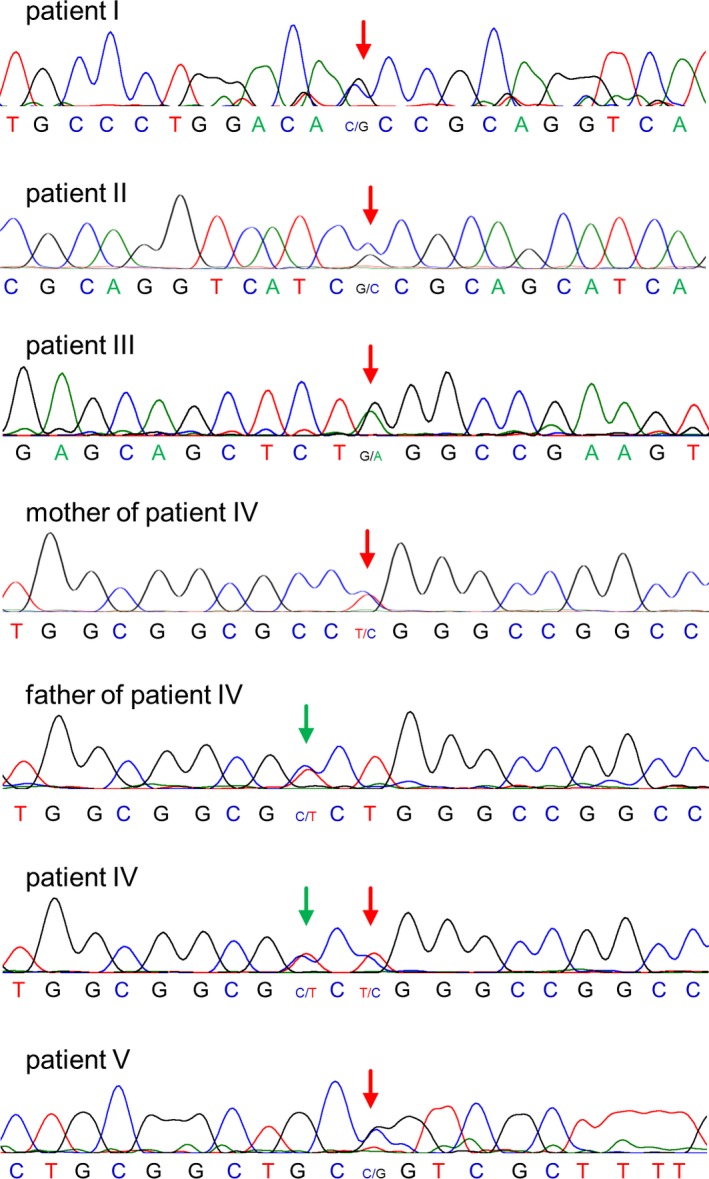
Molecular findings based on Sanger‐sequencing in five unrelated MPS‐I patients. Exemplary sequence traces showing the hereozygous presence of five pathogenic *IDUA* variants (red arrow) in DNA from index patients (I‐V) as well as in DNA from mother of patient IV. Father of patient IV carries a benign single nucleotide variant rs115929690 (green arrow) sugesting that patient IV has inherited a pathogenic variant from his mother and a benign variant from his father, respectively

All patients enrolled in this study had a clinical diagnosis of MPS I, but Sanger‐sequencing performed in a routine diagnostic setting had only detected a single heterozygous *IDUA* mutation. By repeating Sanger‐sequencing of the whole gene, we confirmed the presence of the previously reported heterozygous variants (Figure 1), and the lack of additional, potentially pathogenic variants (data not shown). The five mutations comprised the three missense variants c.979G>C (p.A327P), c.1469T>C (p.L490P) and c.1598C>G (p.P533R), and the nonsense variant c.1205G>A (p.W402X), all of which have been found in patients previously (Bunge et al., 6; Scott, Litjens, Hopwood, & Morris, 16; Scott, Litjens, Nelson et al., 17; Tieu, Bach, Matynia, Hwang, & Neufeld, 19), as well as the splice site variant c.973‐7C>G (p.?), which is reported here for the first time. The splice site variant was further analyzed by in silico prediction tools which strongly suggested mis‐splicing of exon 8 (p.?) to be the primary consequence (Supporting Information Table S2).

### 3.2 Application of a previously introduced, IDUA‐specific MLPA assay suggests the presence of a heterozygous deletion in one patient and the presence of a heterozygous duplication in another

An *IDUA*‐specific MLPA assay as presented by us previously (Breen et al., 4) suggested normal diploid *IDUA* copy number in three of the five patients. In the sample with the heterozygous c.1469T>C variant, the signal for a 3'UTR‐specific probe was reduced by ~50%, while in the sample with the heterozygous c.1598C>G variant, the signals for exons 2, and 7 were increased by ~50% (data not shown). These observations suggested the heterozygous presence of one presumably large deletion and one presumably large duplication, respectively.

### 3.3 Additional MLPA probes confirm the two suspected IDUA CNVs, define their extent, and characterize the remaining three samples as definitely CNV‐negative

**Figure 2:**
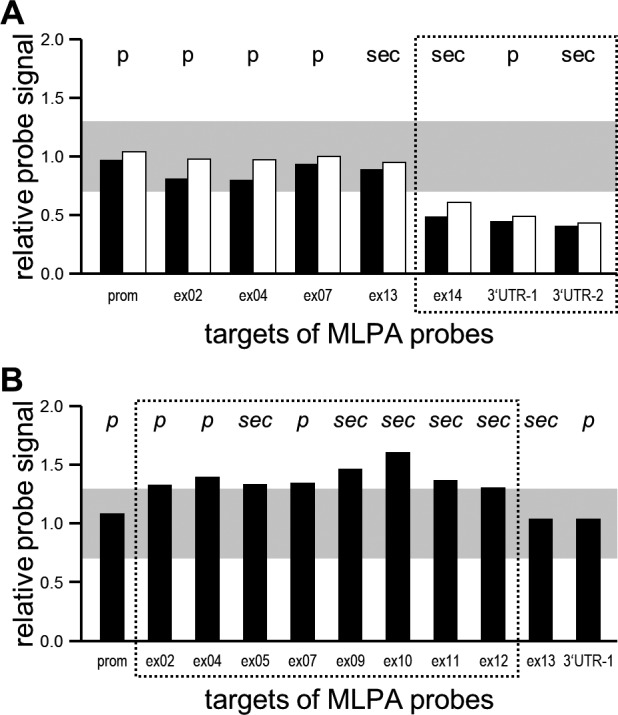
*IDUA*‐specific MLPA findings for CNV‐positive patients. (a) Reduced relative probe signals for exon 14 and both 3’UTR probes indicate a heterozygous deletion in the index case “NOR” (black; patient IV) and her father (white). (b) Increased relative probe signals for exons 2 to 12 indicate a heterozygous duplication in index case “Manch TP” (patient V). p, primary MLPA probe derived from our previously published probe set; sec, secondary MLPA probe added in the frame of the present study; gray box, signal range (0.7–1.3) that is considered to indicate presence of two genomic copies; stippled boxes, deduced (minimal) range of the CNVs

Based on the above described suggestive observations, we designed additional MLPA probes for exons not covered by our initial assay, and applied them to the two samples of interest. A second 3'UTR‐specific probe confirmed the deletion in sample with the heterozygous c.1469T>C variant. Two novel probes which target the penultimate exon 13 and a sequence near the stop codon in exon 14, respectively, revealed that the deletion was restricted to exon 14, and that it overlapped with the coding sequence. Its 3’‐extend, however, could not be delineated by the set of MLPA probes used (Figure 2a). The duplication as suggested by the increased MLPA signals for exons 2 and 7 was first confirmed by a novel MLPA probe against exon 5. Based on additional probes, its 3’‐extend was subsequently mapped to intron 12 (Figure 2b). The application of all novel MLPA probes to the three samples that had remained negative upon analysis with the original MLPA assay did still not reveal evidence for *IDUA* CNVs (data not shown).

## 4 DISCUSSION

This study reports on a heterozygous deletion and a heterozygous duplication which partially affect the *IDUA*. Several lines of evidence support pathogenicity of these CNVs. First, they are not listed by variation databases (ExAC, database of genomic variants, Decipher). Second, they were identified in MPS I patients in whom conventional Sanger‐sequencing had only identified a pathogenic *IDUA* variant on one allele. Third, they are predicted to result in reduced *IDUA* mRNA levels. For the duplication, this is based on the out‐of‐frame nature of the duplicated region, and nonsense‐mediated mRNA decay as the likely consequence (Nagy & Maquat, 11). The deletion, which involves the gene's 3’‐end including some coding nucleotides and the stop codon, should entail nonstop‐mediated mRNA decay (Hamby, Thomas, Cooper, & Chuzhanova, 10; Rebelo et al., 13). By thereby representing bona fide loss‐of‐function alleles, both CNVs thus resemble the majority of already known pathogenic variants in *IDUA* (Bertola et al., 3; Poletto et al., 12). We conclude that the above arguments represent strong cumulative evidence for pathogenicity of both the deletion and the duplication.

We previously presented an MPS I patient who carried two distinct large *IDUA* deletions (Breen et al., 4). Together with the findings presented here, a total of four pathogenic *IDUA* CNVs have now been identified. This number is small compared to the long list of known “small” aberrations [HGMD]. However, a fraction of patients for whom homozygous variants have been reported may in fact be compound heterozygous for this variant and a large overlapping deletion. In addition, MPS I patients with only one identified mutant *IDUA* allele are frequently encountered (Bertola et al., 3; Scott et al., 15; Uttarilli et al., 20). The existence of hitherto undetected *IDUA* CNVs can therefore be expected. The availability of MLPA assays has greatly facilitated the detection of deletions as well as duplications in many other inherited disorders (Günther et al., 9). Our corresponding tool will be made available to researchers interested in more accurately defining the prevalence of *IDUA* CNVs.

We compiled a total of five MPS I samples in which a second pathogenic variant could not be identified. Two of these could eventually be explained by pathogenic CNVs, while for three samples a second mutation remains to be discovered. One may therefore hypothesize that undetected/unscreened variants in the promotor, in other regulatory elements or in deep intronic regions (Beesley et al., 2; Vazna et al., 21) and/or large genomic inversions (Scott, Litjens, Nelson et al., 17; Scott et al., 18) contribute to the mutational spectrum in the *IDUA*. More generalized approaches such as whole genome sequencing will be required to eventually complete the mutational spectrum in *IDUA*.

## CONFLICT OF INTEREST

The authors declare no conflict of interest.

## AUTHOR CONTRIBUTIONS

Amir Jahic: design and conceptualization of the study, analysis and interpretation of the data, drafting and revising of the manuscript for intellectual content. Sven Günther: analysis and interpretation of the data, drafting and revising of the manuscript for intellectual content. Nicole Muschol: interpretation of the data, revising of the manuscript for intellectual content. Barbro Fossøy Stadheim: interpretation of the data, revising of the manuscript for intellectual content. Øivind Braaten: interpretation of the data, revising of the manuscript for intellectual content. Hanne Kjensli Hyldebrandt: interpretation of the data, revising of the manuscript for intellectual content. Gé‐Ann Kuiper: interpretation of the data, revising of the manuscript for intellectual content. Karen Tylee: interpretation of the data, revising of the manuscript for intellectual content. Frits A. Wijburg: interpretation of the data, revising of the manuscript for intellectual content. Christian Beetz: design and conceptualization of the study, analysis and interpretation of the data, drafting and revising of the manuscript for intellectual content
