# Supplementary material for: “Missing mutations” in MPS I: Identification of two novel copy number variations by an IDUA‐specific in house MLPA assay
Source: Mol Genet Genomic Med. 2019 Jul 18;7(9):e00615. doi: 10.1002/mgg3.615 (PMC6732313; doi:10.1002/mgg3.615)
Supplement: Supplementary file 1 [file MGG3-7-e00615-s001.docx]

**Supplementary Table 1A.** Sequences for the *IDUA*-specific MLPA kit.

| **MLPA probe** | **sequence of corresponding oligonucleotide (target-specific sequence in bold, binding sites for universal primers in normal font)** |
| --- | --- |
| IDUA promotor left half-probe | GGGTTCCCTAAGGGTTGGA**CCCGCAAGGAAGCGGGGCTCCA** |
| IDUA exon02 left half-probe | GGGTTCCCTAAGGGTTGGA**CCGTCCCTCACCGCGGCATCA** |
| IDUA exon04 left half-probe | GGGTTCCCTAAGGGTTGGA**CCTCGGGCCACTTCACTGACTTTGAGGACA** |
| IDUA exon05 left half-probe | GGGTTCCCTAAGGGTTGGA**CGTGGAATGAGCCAGACCACCACGACTTTGACA** |
| IDUA exon07 left half-probe | GGGTTCCCTAAGGGTTGGA**CCCGCAGGGTGCGCGCAGCTCCATCTCCATCCTGGAGCAGGAGA** |
| IDUA exon09 left half-probe | GGGTTCCCTAAGGGTTGGA**CCGAAGTGTCGCAGGCCGGGACCGTCCTGGACAGCA** |
| IDUA exon10 left half-probe | GGGTTCCCTAAGGGTTGGA**CCTGGTCTACGTCACGCGCTACCTGGACA** |
| IDUA exon11 left half-probe | GGGTTCCCTAAGGGTTGGA**CGCCCCGAGAAGCCGCCCGGGCAGGCA** |
| IDUA exon12 left half-probe | GGGTTCCCTAAGGGTTGGA**CCACGTGTTCATCCGACCAGACCAGA** |
| IDUA exon13 left half-probe | GGGTTCCCTAAGGGTTGGA**CCCCGGTCAGCAGGAAGCCATCGACCTTCAACCTCTTTGTGT** |
| IDUA exon14 left half-probe | GGGTTCCCTAAGGGTTGGA**CCAAGAGGGCCCCCATCCCCGGGCA** |
| IDUA 3’UTR first left half-probe | GGGTTCCCTAAGGGTTGGA**CCAGCTGGAGCGAGGCCTCTTTCCCCCTCGCTCCCACTCAGACCACCCCCA** |
| IDUA 3’UTR second left half-probe | GGGTTCCCTAAGGGTTGGA**CCCTTGCTTTCTTACTTGTTCTGCAGAGCGGGGAGCTGAGGCTGCATGAGGGA** |
|  |  |
| IDUA promotor right half-probe | **AGCCCTGCCGTGCTCCCGG**TCTAGATTGGATCTTGCTGGCAC |
| IDUA exon02 right half-probe | **AGCAGGTCCGGACCCACTGGCTGCTGG**TCTAGATTGGATCTTGCTGGCAC |
| IDUA exon04 right half-probe | **AGCAGCAGGTGTTTGAGTGGAAGGACTTGG**TCTAGATTGGATCTTGCTGGCAC |
| IDUA exon05 right half-probe | **ACGTCTCCATGACCATGCAAGGTGTGCACCGCTTCCTGG**TCTAGATTGGATCTTGCTGGCAC |
| IDUA exon07 right half-probe | **AGGTCGTCGCGCAGCAGATCCGG**TCTAGATTGGATCTTGCTGGCAC |
| IDUA exon09 right half-probe | **ACCACACGGTGGGCGTCCTGGCCAGCG**TCTAGATTGGATCTTGCTGGCAC |
| IDUA exon10 right half-probe | **ACGGGCTCTGCAGCCCCGACGGCGAGTG**TCTAGATTGGATCTTGCTGGCAC |
| IDUA exon11 right half-probe | **AGTGGCAGTCCCCTAACCCGCGCCGCGG**TCTAGATTGGATCTTGCTGGCAC |
| IDUA exon12 right half-probe | **ACCAGCTGCCCTTGGGTCAGGGGCAGGGCG**TCTAGATTGGATCTTGCTGGCAC |
| IDUA exon13 right half-probe | **TCAGCCCAGGTGCGCCCACCACCCGCTGCCCTGGACTCGG**TCTAGATTGGATCTTGCTGGCAC |
| IDUA exon14 right half-probe | **ATCCATGAGCCTGTGCTGAGCCCCAGTGG**TCTAGATTGGATCTTGCTGGCAC |
| IDUA 3’UTR first right half-probe | **AGCCTGGACTGGAAGTGTGTTGAGCCCCTGGGTCAGG**TCTAGATTGGATCTTGCTGGCAC |
| IDUA 3’UTR second right half-probe | **AGACTCCCGTACCCCAGCACGGTGG**TCTAGATTGGATCTTGCTGGCAC |

**Supplementary Table 1B.** Product sizes of the *IDUA*-specific MLPA kit.

| **MLPA probe** | **size of MLPA product [bp]** |
| --- | --- |
| *IDUA promotor* | 083 |
| *IDUA* exon 02 | 090 |
| *IDUA* exon 04 | 102 |
| *IDUA* exon 05 | 114 |
| *IDUA* exon 07 | 109 |
| *IDUA* exon 09 | 105 |
| *IDUA* exon 10 | 099 |
| *IDUA* exon 11 | 097 |
| *IDUA* exon 12 | 098 |
| *IDUA* exon 13 | 124 |
| *IDUA* exon 14 | 112 |
| *IDUA* 3’UTR first | 130 |
| *IDUA* 3’UTR second | 120 |
