# Supplementary material for: “Missing mutations” in MPS I: Identification of two novel copy number variations by an IDUA‐specific in house MLPA assay
Source: Mol Genet Genomic Med. 2019 Jul 18;7(9):e00615. doi: 10.1002/mgg3.615 (PMC6732313; doi:10.1002/mgg3.615)
Supplement: Supplementary file 2 [file MGG3-7-e00615-s002.docx]

**Supplementary Table 2:** *In silico* analysis of the novel splice site variant c.973-7C>G in *IDUA*, detected in this study.

This table shows only relevant results related to the mutation position and context.

The mutation occurs in the late intronic positions, the following table show results of acceptor splice sites that could be affected by the mutation

| **Predicted signal** | **Prediction algorithm** | **cDNA Position** | **Interpretation** |
| --- | --- | --- | --- |
| New Acceptor Site | 1 - HSF Matrices | 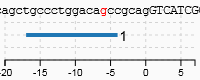 | **Activation of an intronic cryptic acceptor site. Potential alteration of splicing.** |
| Broken WT Acceptor Site | 1 - MaxEnt | 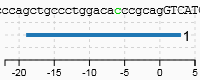 | **Alteration of the WT acceptor site, most probably affecting splicing.** |

[IDUA](http://grch37.ensembl.org/Homo_sapiens/geneview?gene=ENSG00000127415) Gene > [ENST00000247933](http://grch37.ensembl.org/Homo_sapiens/transview?db=core;transcript=ENST00000247933) Transcript > Exon number: 8 (217 bp) + 100 intronic nucleotides at exon ends is/are analyzed by Human Splicing Finder (HSF); WT: Wild Type.
